# Functional screening of a Caatinga goat (*Capra hircus*) rumen metagenomic library reveals a novel GH3 β-xylosidase

**DOI:** 10.1371/journal.pone.0245118

**Published:** 2021-01-15

**Authors:** Betulia de Morais Souto, Ana Carolina Bitencourt de Araújo, Pedro Ricardo Vieira Hamann, Andrêssa de Rezende Bastos, Isabel de Souza Cunha, Julianna Peixoto, Ricardo Henrique Kruger, Eliane Ferreira Noronha, Betania Ferraz Quirino

**Affiliations:** 1 Genetics and Biotechnology Laboratory, Embrapa-Agroenergy, Brasília, DF, Brazil; 2 Department of Cellular Biology, Laboratory of Enzymology, Universidade de Brasília, Brasília, DF, Brazil; 3 Genomic Sciences and Biotechnology Program, Universidade Católica de Brasília, Brasília, DF, Brazil; Dong-A University, REPUBLIC OF KOREA

## Abstract

Functional screening of metagenomic libraries is an effective approach for identification of novel enzymes. A Caatinga biome goat rumen metagenomic library was screened using esculin as a substrate, and a gene from an unknown bacterium encoding a novel GH3 enzyme, BGL11, was identified. None of the BGL11 closely related genes have been previously characterized. Recombinant BGL11 was obtained and kinetically characterized. Substrate specificity of the purified protein was assessed using seven synthetic aryl substrates. Activity towards nitrophenyl-β-D-glucopyranoside (pNPG), 4-nitrophenyl-β-D-xylopyranoside (pNPX) and 4-nitrophenyl-β-D-cellobioside (pNPC) suggested that BGL11 is a multifunctional enzyme with β-glucosidase, β-xylosidase, and cellobiohydrolase activities. However, further testing with five natural substrates revealed that, although BGL11 has multiple substrate specificity, it is most active towards xylobiose. Thus, in its native goat rumen environment, BGL11 most likely functions as an extracellular β-xylosidase acting on hemicellulose. Biochemical characterization of BGL11 showed an optimal pH of 5.6, and an optimal temperature of 50°C. Enzyme stability, an important parameter for industrial application, was also investigated. At 40°C purified BGL11 remained active for more than 15 hours without reduction in activity, and at 50°C, after 7 hours of incubation, BGL11 remained 60% active. The enzyme kinetic parameters of *K*_m_ and *V*_max_ using xylobiose were determined to be 3.88 mM and 38.53 μmol.min^-1^.mg^-1^, respectively, and the *K*_cat_ was 57.79 s^-1^. In contrast to BLG11, most β-xylosidases kinetically studied belong to the GH43 family and have been characterized only using synthetic substrates. In industry, β-xylosidases can be used for plant biomass deconstruction, and the released sugars can be fermented into valuable bio-products, ranging from the biofuel ethanol to the sugar substitute xylitol.

## 1. Introduction

Hemicellulose is the second most highly abundant polysaccharide in lignocellulosic biomass. Unlike cellulose, it is not homogeneous, being composed of pentoses (e.g., xylose, arabinose), hexoses (e.g., mannose, glucose, galactose), and sugar acids [1–3]. One type of hemicellulose is xylan, which is β-1,4-linked xylopyranosyl units often modified by acetyl, arabinofuranosyl and glucuronyl groups [1, 4]. Xylan can be covalently linked to lignin by aromatic esters [5]. Endo-1,4-β-xylanases (EC 3.2.1.8), which cleave the xylan backbone, and β-D-xylosidases (EC 3.2.1.37), which act on xylobiose to release two xylose monomers, are two fundamental enzymes for the degradation of hemicellulose. However, for its complete degradation, several accessory enzymes are also needed, such as exo-oligoxylanases, α-arabinofuranosidases, and α-glucuronidases, as well as esterases, acetyl xylanesterases and feruloyl esterases [5].

The culture-independent metagenomic approach is a powerful method to identify new biocatalysts, particularly by functional screens, which are not sequence-biased. Enzymes can operate under mild conditions being considered eco-friendly, and thus have great appeal for green industrial applications [6, 7]. In addition, the recent growth of synthetic biology has increased the need for biochemically characterized enzymes. To obtain higher yields of a bioproduct, pathway fine-tuning is often needed. This can be achieved by gene swapping so that the same reaction is catalyzed by an enzyme with more favorable biochemical properties. Therefore, enzyme discovery through a functional metagenomic approach, followed by biochemical characterization is crucial to link gene sequence to practical applications in industry.

β-D-xylosidases hydrolyze the disaccharide xylobiose and xylooligosaccharides. They are also able to hydrolyze (1→4)-β-D-xylans by removing successive D-xylose residues from the non-reducing end [8, 9]. There are few biochemically characterized β-xylosidases in the literature. The Carbohydrate Active Enzyme (CAZy) database (http://www.cazy.org/) classifies glycoside hydrolases (GH) into families based on amino acid sequence similarity. According to CAZy, β-D-xylosidase activity (EC 3.2.1.37) is found in glycoside hydrolase families GH1, 2, 3, 5, 10, 30, 39, 43, 51, 52, 54, 116, and 120 [10]. In terms of catalytic mechanism, β-xylosidases can be classified according to the stereochemical configuration at the anomeric carbon as a two-step double (retaining) or a one-step single displacement (inverting) reaction [5, 11].

Brazil is known for its unparallel ability to produce plant biomass economically, and with efficient logistics. Because readily available, cheap, and abundant plant biomass is critical for bio-based industries, this will likely open opportunities for Brazil as the new bioeconomy develops. Enzyme discovery aimed at plant biomass deconstruction can take advantage of environments such as the goat rumen, whose microbiota evolved for this particular process [12]. The solid-associated rumen microbiota of free ranging goats from the semiarid Caatinga Brazilian biome has not yet been used for this purpose. To identify new glycoside hydrolases, a Caatinga goat rumen metagenomic library was functionally screened using esculin as substrate. Here we report the identification of a gene from an unknown bacterium named *BGL11*. BGL11 belongs to the GH3 family, and its biochemical characterization as a multifunctional enzyme with high β-xylosidase activity, towards the natural substrate xylobiose, is reported here for the first time.

## 2. Material and methods

### 2.1. Functional screening of a metagenomic library

The coding gene for BGL11 was identified in a functional screen of a goat (*Capra hircus*) rumen metagenomic library from the Brazilian goat breed Moxotó, commonly found in semiarid habitats. Microbial DNA was isolated from the solid-associated fraction of the rumen contents and used to construct a metagenomic library [12, 13]. The microbial DNA was extracted as described in Martin et al. (1994) [14] and Handelsman et al. (1998) [15]. DNA was digested with *Pst*I and DNA fragments in the range of 3 to 8 kb were selected from a gel and cloned into the low expression vector pCF430 [16]. Expression of DNA fragments cloned into pCF430 was induced by arabinose [16]. *E*. *coli* strain EPI300 (Epicentre, USA) was transformed with the microbial DNA, and the resulting metagenomic library was kept at -80°C until screening. A total of 10,839 goat rumen metagenomic library clones were screened on plates containing Luria-Bertani (LB) agar medium supplemented with 0.05% (w/v) iron (III) citrate and 0.01% (w/v) esculin hydrate, tetracycline 5 μg/mL and 0.02% (w/v) arabinose (concentrations of esculin were modified from Difco™ Esculin Iron Agar).

Positive clone activity was further tested in a plate overlay assay using 2 mM 4-methylumbelliferyl-D-glucopyranoside (MUG) dissolved in citrate buffer (50 mM, pH 7.0) in 2% (w/v) agarose. Enzymatic activity was detected as fluorescence by methylumbelliferyl release from MUG after plate exposure to UV light [17]. To confirm positive clone activity, DNA was extracted and retransformed into EPI300 *E*. *coli* and both the esculin and the MUG plate assay were repeated as previously described.

### 2.2. Sequencing and *in silico* analysis of BGL11

DNA from one retransformed positive clone was sequenced by the Sanger method using a primer walking strategy. The open reading frames (ORFs) present in this clone were identified by ORF Finder (NCBI) using standard and alternative genetic codes [18]. The deduced amino acid sequences were analyzed on the NCBI web server using Blastp [19]. Prokaryote promoter predictions were performed using BPROM [20], PePPER prokaryotes promoter [21] and Neural Network Promoter Prediction [22]. Prokaryote terminators were identified using ARNold [23] and FindTerm [20]. ProtCompB software was used to predict the subcellular localization of the BGL11 protein (Softberry, Inc., Mount Kisco, NY - http://www.softberry.com). Putative signal peptides were identified using softwares Signal-BLAST, using gram-negative bacteria as choice into program option [24] and SignalP 5.0 [25]. Geneious software was used to compare the conserved domains present in **BGL11** (this study), *Caulobacter crescentus* **XynB5** (Genbank ID CCNA03149), *Corynebacterium alkanolyticum* **xylD** (Genbank ID AJY53618.1) and Metagenome Rumen from Chinese yaks **RuBGX1** (Genbank ID GQ324952.1). The databases Pfam, PHOBIUS, PRINTS, SIGNALP_EUK, SMART and Superfamily were used in this analysis.

Molecular phylogenetic analysis using the maximum likelihood method was conducted using MEGA version 6 [26] based on the JTT matrix-based model [27]. Reliability of phylogenetic reconstruction was estimated by boot-strapping values (1,000 replicates). The multiple alignment of sequences used in MEGA6 was obtained using Clustal W with standard parameters [28]. Two phylogenetic trees were constructed; one using the BGL11 30 closest protein sequences obtained with BlastP, and another one using BGL11 plus 34 β-xylosidases characterized kinetically from the scientific literature.

Molecular mass, isoelectric point (pI) including the 6 X His-tag, and extinction coefficient of BGL11 were predicted using ProtParam tool (Expasy) [29].

### 2.3. PCR amplification and cloning

The full-length gene *BGL11* (2,447 bp) was amplified by polymerase chain reaction (PCR) using plasmid DNA from there transformed positive clone using primers BGL11pETNde_F (5’ CAT ATG AGA GGA TTC ATT ATG ACG TGC 3’) and BGL11pETXhoI_R (5’ CTC GAG ATT CGT AAT TGT TAC CTC CG 3’). The amplification reaction was carried out using Platinum® *Taq*DNA Polymerase High Fidelity (Life Technologies), 10 μM of each primer using the following protocol: 1 cycle at 94°C for 2 minutes, 35 cycles of denaturation at 94°C for 30 seconds, annealing at 57°C for 30 s, and extension at 68°C for 3 minutes and a final extension at 68°C for 7 minutes. The amplified DNA fragment was purified from a 1% agarose gel and cloned into pGEM®-T Easy vector (Promega), following the manufacturer’s instructions. The cloned fragment was then transferred to the expression vector pET21a(+) (Novagen®) using *Xho*I and *Nde*I restriction sites, which lead to the addition of a 6 X histidine tag to the BGL11 C-terminus. The resulting construct, pET21BGL11, was used to transform *E*. *coli* BL21(DE3) electro-competent cells [30]. The transformed bacterial cells were plated on LB medium agar supplemented with esculin, iron citrate, ampicillin, as previously described, and incubated at 37°C overnight. Colonies displaying a dark halo were stored at -80°C in glycerol 20%, and used in protein expression experiments.

### 2.4. Protein expression and purification

The autoinduction method was used for protein expression [31]. After incubation at 37°C to obtain an optical density of approximately 1.0, the culture was incubated for approximately 48 hours at 18°C and 250 rpm. Cells were harvested and the culture medium was stored. BGL11 has a peptide signal and was secreted by the transformed *E*. *coli* BL21(DE3). BGL11 was purified from the medium, using immobilized metal affinity chromatography (IMAC). An equilibrated affinity column pre-packed with Ni Sepharose High Performance (HisTrap HP 1 or 5 mL column–GE Healthcare) was injected with 100 mL of BGL11 medium culture. The column was washed with 10 volumes of column of LEW buffer (Protino®) pH 8.0 containing 10 mM imidazole. Protein elution was conducted in three steps: 3 volumes of LEW buffer pH 8.0 containing 50 mM imidazole; 2 volumes of LEW Buffer pH 8.0 containing 75 mM imidazole, and finally 5 volumes of LEW Buffer pH 8.0 containing 250 mM imidazole. The peaks of the elution step flow through were fractionated into 1 mL tubes. The sample containing the BGL11 protein was desalted, and its buffer exchanged using PD-10 desalting columns (GE Healthcare) packed with Sephadex G-25 medium, following the manufacturer’s protocol.

The purified and desalted BGL11-containing sample was further analyzed by 10% SDS-PAGE and stained with colloidal Coomassie blue G250. Protein concentration was directly measured using a NanoDrop (ThermoFisher Scientific) by method A280, based on the Beer-Lambert law, using the extinction coefficient (ɛ(x1000) M^-1^. cm^-1^) and molecular weight (kilodaltons).

### 2.5. Enzyme assays

BGL11 activity towards different substrates was tested in 100 μL reaction assays containing 5 mM of aryl substrates or 10 mM of disaccharides in buffer MES pH 6.0 at 50°C for 15 min (aryl substrates) or 30 min (disaccharides), in triplicates. The aryl substrates used were 4-nitrophenyl-β-D-glucopyranoside (pNPG), 4-nitrophenyl-β-D-xylopyranoside (pNPX), 4-nitrophenyl-β-D-galactopyranoside (pNPGal), 4-nitrophenyl-β-D-mannopyranoside (pNPM), 4-nitrophenyl-α-D-glucopyranoside (pNPαG), 4-nitrophenyl-β-D-rhamnopyranoside (pNPR), 4-nitrophenyl-β-D-cellobioside (pNPC) and salicin. The disaccharides used were cellobiose, xylobiose, maltose, and lactose. All chemicals were purchased from Sigma Aldrich, except xylobiose which was purchased from Megazyme. After stopping the reaction by addition of 100 μL cold 1 M sodium carbonate, the amount of 4-nitrophenol released from aryl substrates was immediately measured by reading absorbance at 405 nm in a SpectraMax M3 spectrophotometer (Molecular Devices). Data were compared to a standard curve prepared with 4-nitrophenol (pNP) ranging from 0 to 0.07 μmol. The glucose or xylose released from disaccharides hydrolyzes were quantified using a glucose monoreagent kit (Bioclin, Brazil), which is based on the glucose oxidase method (GOD-PAP). After 30 min of reaction time, the enzymatic reaction was stopped by incubation at 95°C for 10 min, and then 150 μL of monoreagent glucose was added. After incubation at 37°C for 10 min, absorbance was measured at 505 nm in a SpectraMax M3 spectrophotometer (Molecular Devices). Data were compared to a standard curve prepared with glucose ranging from 0 to 0.0072 mg or xylose ranging from 0 to 9 mg. One unit of BGL11 activity was defined as the amount of enzyme that released 4-nitophenol or glucose or xylose at a rate of 1 μmol per minute under standard conditions.

Optimal pH for BGL11 activity was assayed using a buffer system of constant ionic strength, the McIlvaine buffer [34]. Enzymatic reactions were performed using 5 mM pNPG as substrate at 40°C for 15 min in 100 mM McIlvaine buffer at pH 4.0, 4.4, 4.8, 5.2, 5.6, 6.0, 6.4, 6.8, and 7.6. BGL11 optimal temperature was determined at the optimal pH of 5.6, incubating reactions for 15 min at temperatures of 20°C, 25°C, 30°C, 35°C, 40°C, 45°C, 50°C, 55°C, 60°C, 65°C, 70°C, and 75°C. Thermostability assays were performed using 200 nM of purified BGL11; residual enzymatic activity after pre-incubation of BGL11 at 40°C and 50°C for up to 15 hours was measured using pNPX as substrate and buffer MES pH 6.0.

The kinetic parameters of K_*m*_, V_*max*_ and K_*cat*_, were determined using 200 nM of purified BGL11 using MES buffer pH 6.0 and incubation time of 30 minutes at 50°C. Xylobiose ranging from 0 to 20 mM was used as substrate. All enzymatic activity measurements were performed in triplicate. Kinetic parameters were calculated using SigmaPlot software from company Systat Software Inc. The data were fitted to Michaelis-Menten enzyme kinetics model, and the curve with best coefficient of determination (R^2^) was used to determine *K*_m_ and *V*_max_. Adjusted values were used to plot a non-linear curve (Michaelis-Menten) and a linearized model (Lineweaver-Burke).

### 2.6. Accession number

The BGL11 nucleotide sequence (i.e. not codon-optimized) was deposited at GenBank database under GenBank accession number MN661154.

## 3. Results

### 3.1. Screening and *in silico* analysis of BGL11

**Fig 1:**
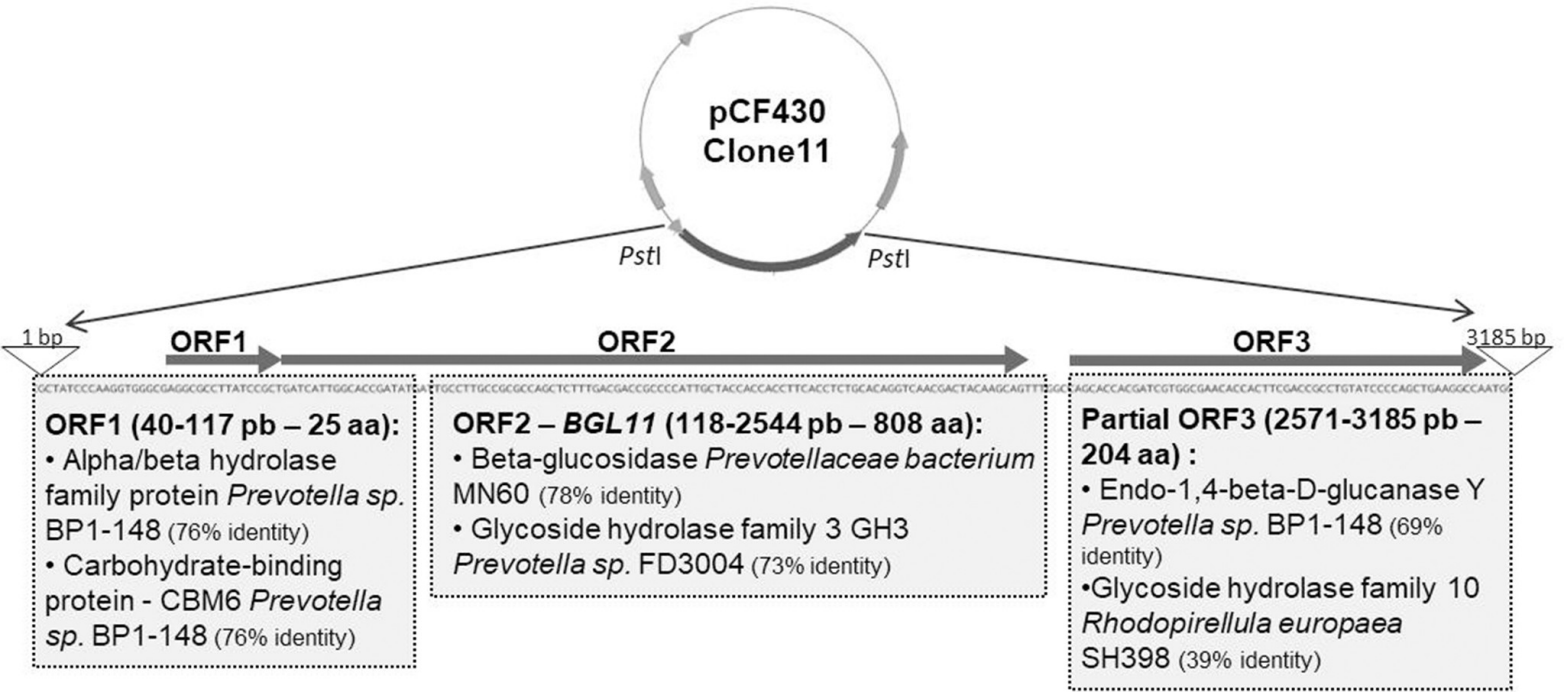
Schematic of Clone 11 insert obtained in a functional screen of a goat rumen metagenomic library for hydrolysis of the glucoside esculin. The Clone 11 insert has 3,185 bp, and a total of 3 ORFs related to carbohydrate metabolism are present; ORF1 and 2 are complete and ORF3 is partial. Results for Blastp analysis of each ORF are shown in gray boxes. ORF2 (118 to 2,544 bp), which showed similarity to a beta-glucosidase from an unknown *Prevotella ceaebacterium*, was named *BGL11* and chosen to be investigated.

Functional screening of a goat rumen metagenomic library identified three positive clones that displayed a dark halo on petri plates containing LB medium supplemented with esculin and iron citrate. Clone 11 showed a larger halo than other positive clones (S1 Fig). Sequencing and analysis of its 3,185 bp insert, revealed two complete ORFs (open reading frame), and one partial ORF related to carbohydrate metabolism (Fig 1).

According to BlastP analysis, ORF1 encodes a small peptide with only 25 amino acids long, 76.32% identical and 84% similar to an alpha/beta hydrolase family protein from *Prevotella* sp. BP1-148 (Genbank ID SDG26698.1). ORF1 also showed 76.32% identity and 84% similarity to a carbohydrate-binding protein (CBM) from *Prevotella* sp. BP1-148 (NCBI Reference Sequence: WP_091814266.1).

Analysis of the complete ORF2 identified the encoded protein as a member of the GH3 family of carbohydrate active enzymes. It is 78% identical to a beta-glucosidase from a *Prevotellaceae bacterium*, as well as 73% identical and 84% similar to a Glycoside Hydrolase Family 3 protein from *Prevotella* sp. (FD3004 NCBI Reference Sequence: WP_036910324.1), and it was named BGL11. BGL11 was also analyzed for the presence of conserved domains and seven of these were identified (Table 1).

**Table 1:** Conserved domains present in BGL11.

Domain name	Accession	Description	Amino acids	E-value
**BglX**	COG1472	Periplasmicβ-glucosidase and related glycosidases;	35–435	5.95e-59
**Glyco_hydro_3_C**	pfam01915	Glycosyl hydrolase family 3 C-terminal domain;	398–666	3.44e-58
**Glyco_hydro_3**	pfam00933	Glycosyl hydrolase family 3 N-terminal domain;	87–354	1.15e-53
**Fn3-like**	pfam14310	Fibronectin type III-like domain;	698–771	5.52e-16
**PRK05337**	PRK05337	β-hexosaminidase; Provisional	125–283	1.04e-09
**PRK15098**	PRK15098	β-D-glucoside glucohydrolase; Provisional	9–787	2.67e-107
**PLN03080**	PLN03080	Probable β-xylosidase; Provisional	102–738	1.32e-52

Blastp analysis of ORF3, which is incomplete, revealed a 69.15% identity and 79% similarity to endo-1,4-β-D-glucanase Y of *Prevotella* sp. BP1-148 (Genbank ID: SDG13112.1). It also showed 39.23% identity, and 54% similarity to a family 10 glycoside hydrolase from *Rhodopirellula europaea* SH398 (Genbank ID: EMI26865.1).

The BGL11 ORF was also compared to the *Prevotella ruminicola* 23 complete genome (gb|CP002006.1) using Blastp. BGL11 showed 69% identity and 81% similarity to a GH3 ORF PRU_2312 (ADE81919.1). The *BGL11* gene was also aligned with PRU_2312 in Geneious software, showing 67% identity. Analysis of the region surrounding PRU_2312, identified genes involved in carbohydrate degradation on both sides: GH2—PRU_2313 (ADE82711.1), alfa-glucosidase- PRU_2314 (ADE81436.1), putative glycosyl hydrolase -PRU_2308 (ADE82976.1) and putative polygalacturonase/ beta-xylosidase -PRU_2307 (ADE83743.1).

Analysis of the Clone 11 insert using the promoter prediction software Bprom identified six putative promoters, but only one located upstream the BGL11 initiation codon, with the -10 box region located at site 119 and the -35 box region at site 100. A similar analysis using the PePPER prokaryotes promoter software identified seven promoters, two of them located upstream the BGL11 initiation codon, p1 starting at site 74 and p2 at site 94. The last software used, Neural Network Promoter Prediction, identified 3 promoters upstream the BGL11 initiation codon starting at 70, 79 and 93 positions. No terminator was identified in Clone 11 using three different terminator prediction programs.

*In silico* analysis of the protein encoded by *BGL11*, estimated a molecular mass of 89,978.1 Daltons (including the 6 x His-tag) and a theoretical isoelectric point (pI) of 5.26. A signal peptide was identified by Signal-BLAST software, with a putative cleavage site after BGL11 amino acid 16 and 43% identical to GFCA_ECOLI, from threonine-rich inner membrane protein gfcA (UniProt acession number P75885). The Software SignalP 5.0 predicts a signal peptide with a cleavage site between positions 15 and 16: LAG-CQ (probability: 0.9957). The signal peptide type was the Sec/SPII: lipoprotein signal peptide transported by the Sec translocon and cleaved by Signal Peptidase II (Lsp). ProtCompB software predicted BGL11 to be secreted.

**Fig 2:**
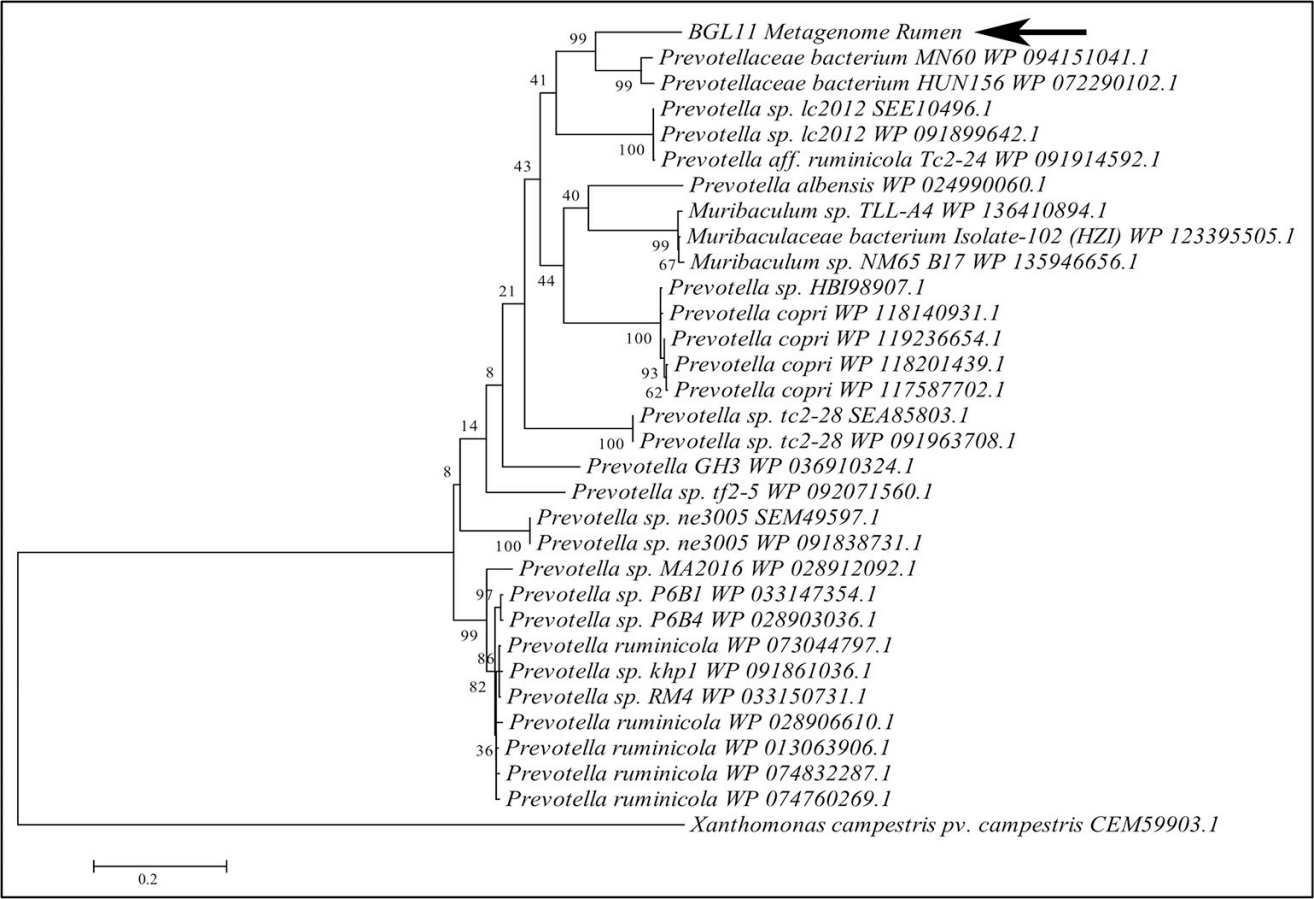
Molecular phylogenetic tree of BGL11 obtained with the software MEGA6, using the maximum likelihood method and bootstrap analysis (1,000 replicates). The percentage of trees in which the associated taxa clustered together is shown next to the branches. In addition to BGL11 (indicated by the arrow), a total of 30 protein sequences similar to BGL11 and retrieved from NCBI are presented in the tree. A beta-glucosidase from *Xanthomonas campestris* (CEM59903.1) was used as outgroup. Each sequence presented shows the species of origin followed by the corresponding Genbank or NCBI identity. Branch lengths are proportional to the number of substitutions per site (bar at the bottom).

A phylogenetic tree constructed with BGL11 and 30 other protein sequences similar to BGL11 retrieved from NCBI is shown in Fig 2. All but three of the most closely related sequences were from *Prevotella* spp. BGL11 clustered with *Prevotellaceae bacterium* MN 60 WP 094151041.1 sequence and *Prevotellaceae bacterium* sequence HUN156 WP 072290102.1; however, it remained in a separate branch. None of the 30 proteins in this phylogenetic tree have been purified or biochemically characterized.

**Fig 3:**
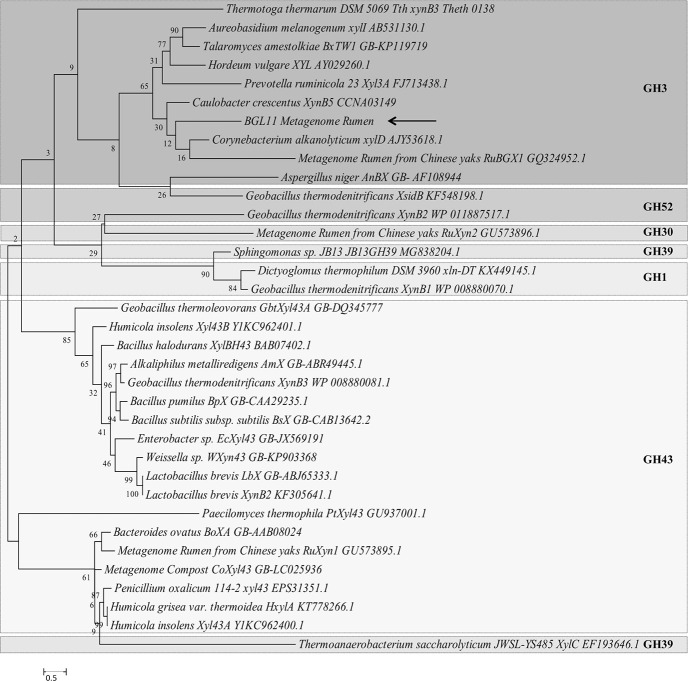
Molecular phylogenetic tree of BGL11 obtained with the software MEGA 6 using the maximum likelihood method and boostrap analysis (1,000 replicates). The percentage of trees in which the associated taxa clustered together is shown next to the branches. In addition to BGL11 (indicated by arrow), the tree shows 34 beta-xylosidase protein sequences whose biochemical characterization has been reported. Each sequence presented shows the species of origin, followed by the protein name, and the Genbank number. Different glycoside hydrolase families (GH1, GH3, GH30, GH39, GH43, GH52) are separated by boxes. Branch lengths are proportional to the number of substitutions per site (bar at the bottom).

**Fig 4:**
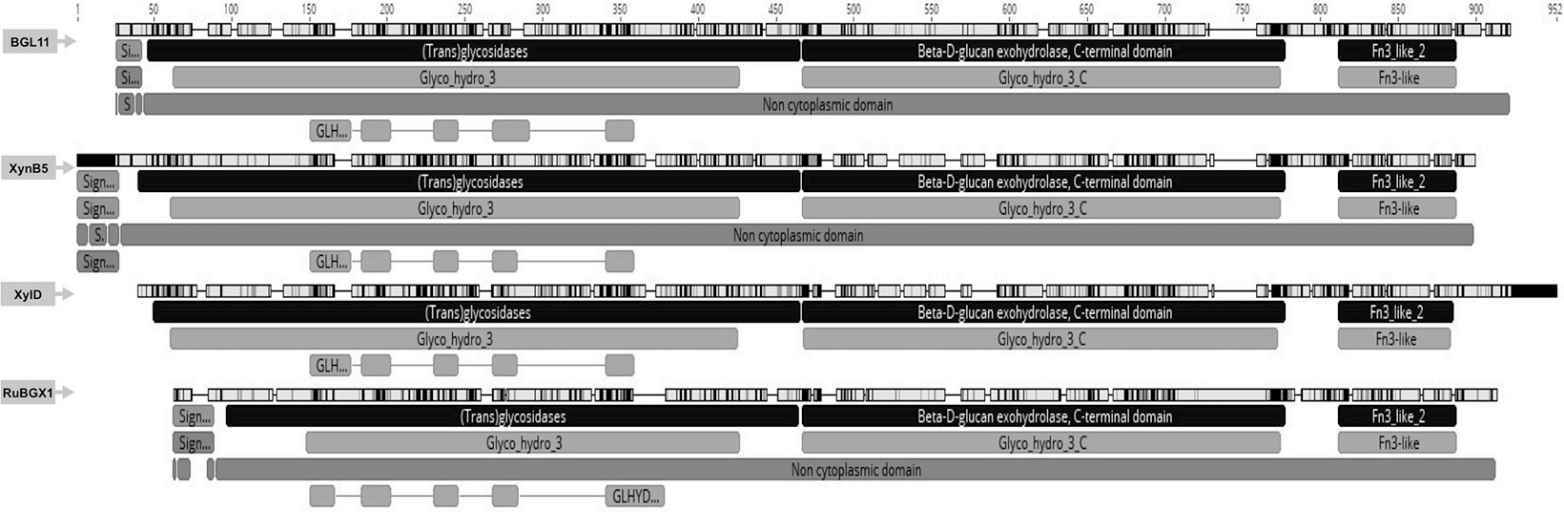
Alignment of BGL11 and the closest phylogetically biochemically characterized related sequences. Sequences used in alignment were: BGL11 (this study), *Caulobacter crescentus* XynB5 (Genbank ID CCNA03149), *Corynebacterium alkanolyticum* xylD (Genbank ID AJY53618.1) and Rumen Metagenome from Chinese yaks RuBGX1 (Genbank ID GQ324952.1) in this order of presentation. Software Geneious was used to generate this picture, using databanks Pfam, PHOBIUS, PRINTS, SIGNALP_EUK, SMART and Superfamily.

To determine the phylogenetic relationship between BGL11 and 34 biochemically characterized β-xylosidases, another phylogenic tree was constructed (Fig 3). Enzymes clustered according to the glycoside hydrolase family, rather than group of origin (i.e., fungi, bacteria, and plant). BGL11 clustered with other GH3 family enzymes. The phylogenetic tree shows that BGL11 is closest to the β-xylosidases XynB5 from *Caulobacter crescentus*, xylD from *Corynebacterium alkanolyticum*, and RuBGX1 from the Chinese yak rumen metagenome. To compare conserved domains, these three β-xylosidase protein sequences were aligned to BGL11 (Fig 4). These four β-xylosidase sequences have a similar pattern of predicted domains. All four sequences have from the Pfam conserved domains Glyco_hydro_3 (PF00933), Glyco_hydro_3_C (PF01915) and Fn3-like (PF14310). The Fn3-like conserved domain was also predicted using the databank SMART (SM01217). Using the Superfamily databank, all sequences showed a (Trans)Glycosidase domain (SSF 51445), and a Beta-D-glucan exohydrolase C-terminal domain (SSF 52279). The domain GLHYDRLASE3 was predicted to be present in all sequences using the PRINTS database (PR00133). Except for xylD from *Corynebacterium alkanolyticum*, all the sequences have predicted signal peptides using databanks SIGNALP_EUK and PHOBIUS.

### 3.2. Enzyme expression, purification, and characterization

**Fig 5:**
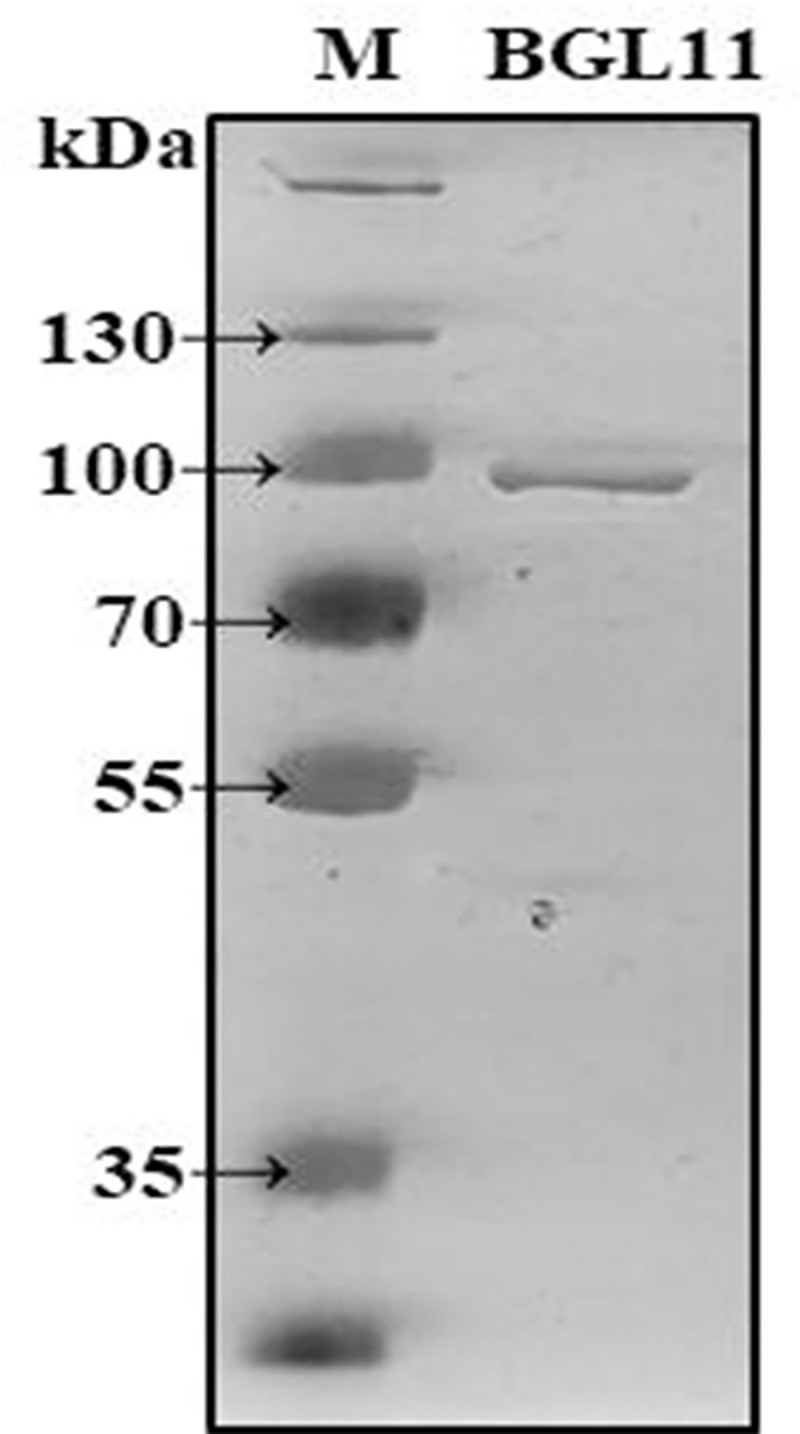
SDS-PAGE analysis of purified BGL11. Lane M, PageRuler™ Plus Prestained 10–250 kDa Protein Ladder (Thermo Scientific™); Lane BGL11, purified protein with the size of 90 kDa.

ORF2 encoding BGL11 was heterologously expressed in *E*. *coli* strain BL21(DE3), and the resulting protein was purified and biochemically characterized. The BGL11 sequence has a predicted peptide signal, and when the autoinduction expression protocol was used, BGL11 protein was found to be secreted to the growth medium. After *E*. *coli* cells were pelleted, BGL11 was purified from the supernatant by immobilized metal affinity chromatography with a Ni-NTA column. As shown in Fig 5, a band of 90 kDa corresponding to BGL11 was obtained.

### 3.3. Enzyme properties

To determine substrate specificity of recombinant BGL11, 12 different aryl-glycoside and disaccharide substrates were tested (Table 2). BGL11 exhibited catalytic activity towards five synthetic substrates (i.e., pNPG, pNPX, pNPGal, pNPC, and pNPαG), as well as four natural substrates (i.e., cellobiose, lactose, salicin, and xylobiose). BGL11 exhibited maximum activity toward xylobiose (0.995687 ± 0.0111 U/mg), and showed no activity toward pNPM, pNPR and maltose.

**Table 2:** BGL11 specific enzymatic activity towards synthetic aryl and natural substrates.

Substrate	U/mg
**pNPG**	0.130008 ± 0.0090
**pNPX**	0.132400 ± 0.0081
**pNPGal**	0.001385 ± 0.0005
**pNPC**	0.083247 ± 0.0026
**pNPαG**	0.003779 ± 0.0015
**pNPM**	0
**pNPR**	0
**Cellobiose**	0.005577 ± 0.0010
**Maltose**	0
**Lactose**	0.002144 ± 0.0001
**Salicin**	0.024972 ± 0.0003
**Xylobiose**	0.995687 ± 0.0111

The aryl substrates used are 4-nitrophenyl-β-D-glucopyranoside (pNPG), 4-nitrophenyl-β-D-xylopyranoside (pNPX), 4-nitrophenyl-β-D-galactopyranoside (pNPGal), 4-nitrophenyl-β-D-mannopyranoside (pNPM), 4-nitrophenyl-α-D-glucopyranoside (pNPαG), 4-nitrophenyl-β-D-rhamnopyranoside (pNPR), 4-nitrophenyl-β-D-cellobioside (pNPC) and salicin; and natural disaccharide substrates used are cellobiose, xylobiose, maltose, and lactose.

**Fig 6:**
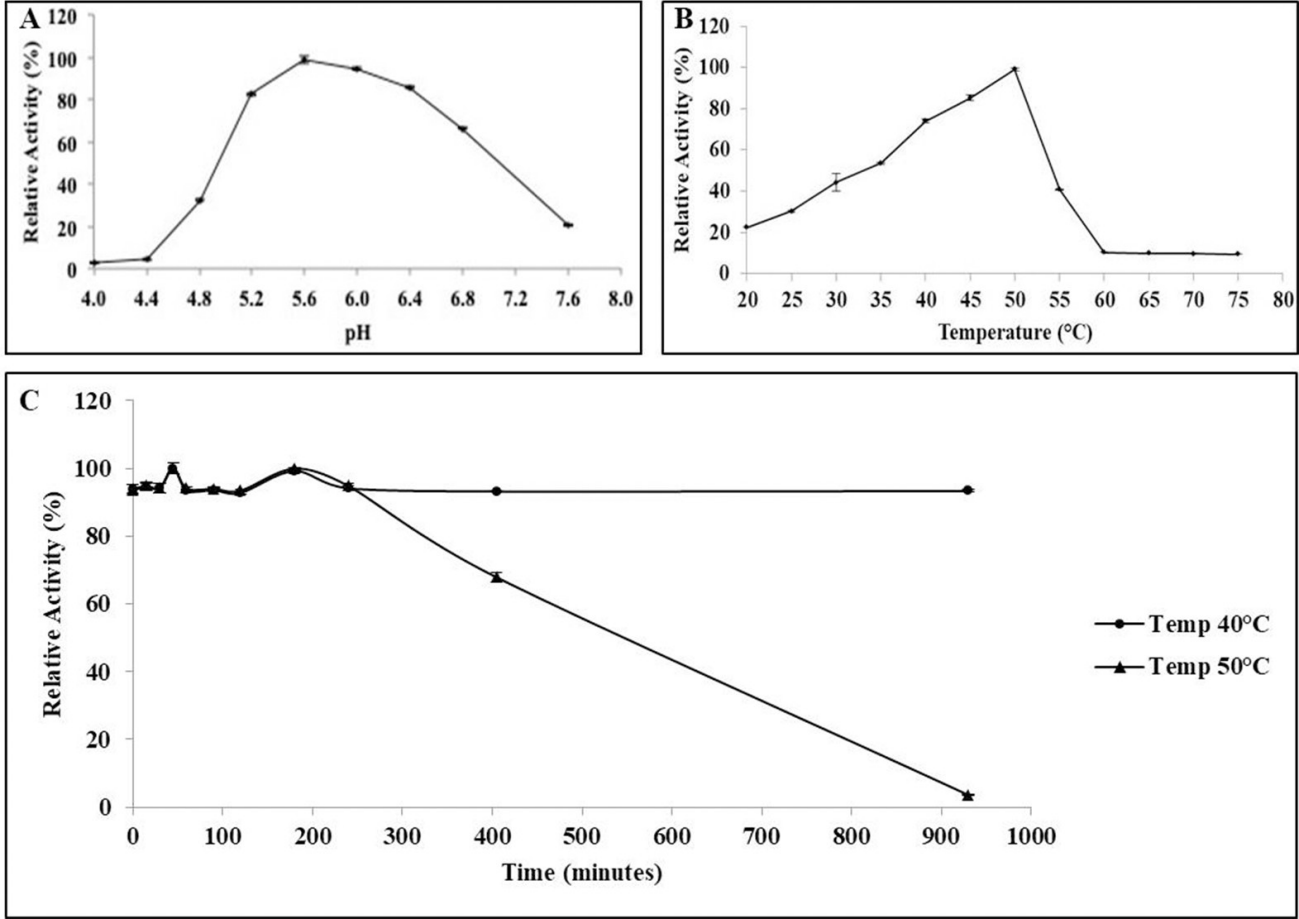
Biochemical characteristics of BGL11. Effects of pH (A), and temperature (B) and thermal stability at 40°C and 50°C (C) of the purified BGL11 relative activity. The assays were performed in triplicate, error bars represent standard deviation.

Optimal pH for BGL11 was determined to be 5.6 (Fig 6); however, BGL11 maintains activity higher than 80% between pH 5.2 and 6.4. Optimal temperature was 50°C, but higher than 80% activity occurred between 43 to 50°C. Enzyme stability was determined at two different temperatures, 40°C and 50°C. BGL11 was more stable at the lower temperature, maintaining activity for more than 15 hours (i.e., 900 minutes). At 50°C, after 7 hours (i.e., 420 minutes) of incubation, BGL11 remained 60% active.

Using xylobiose, kinetic parameters of BGL11 were determined. By non-linear regression analysis, the values of *K*_m_ were calculated as 3.88 ± 0.31 mM, *V*_max_ as 0.0385 ± 0.00084 mmol. min^-1^. mg^-1^, *K*_cat_ as 57.79 s^-1^ and *K*_cat_/*K*_m_ as 14.89 s^-1^. mM^-1^. The R^2^ for the Michaelis-Menten adjustment of data using a non-linear regression model was 0.99 (S2 Fig). For comparison, the kinetic parameters of other β-xylosidases also characterized using the natural substrate xylobiose are shown on Table 3.

**Table 3:** Biochemical parameters of β-xylosidases previously characterized using xylobiose as substrate.

Name (Accession number)	GH	Origin	Species	Opt. Temp.*	Opt. pH**	*K*_m_ (mM)	*V*_max_ (mmol.min^-1^. mg^-1^)	*K*_cat_ (s^-1^)	*K*_cat_/*K*_m_ (s^-1^. mM^-1^)	Ref
**BGL11 (MN661154)**	GH3	Rumen metagenome	Closest hit *Prevotella sp*.	50	5.6	3.88±0.31	0.0385±0.00084	57.79	14.89	This work
**CoXyl43 (LC025936)**	GH43	Compost metagenome	Closest hit *Bacteroides ovatus*	55	7.5	2.02±0.06	-	17.82±0.14	8.82	[32]
**XynB2 (KF305641)**	GH43	Bacterium	*Lactobacillus brevis*	50	6.0	4.8±0.4	0.228±0.008	233	48.54	[33]
**BoXA (AAB08024)**	GH43	Bacterium	*Bacteroides ovatus*	-	-	0.61±0.08	-	69.0±3.7	113.11	[34]
**WXyn43 (KP903368)**	GH43	Bacterium	*Weissella strain* 92	55	6–6.5	7.2±0.5	-	961±25	133.47	[11]
**EcXyl43 (JX569191)**	GH43	Bacterium	*Enterobacter sp*. Closest hit *Enterobacter cloacae*	40	6.0	17.8	-	380	21.34	[35]
**BpX (CAA29235.1)**	GH43	Bacterium	*Bacillus pumilus*	-	-	5.21±0.41	-	38.8±1.4	7.45	[36]
**LbX (ABJ65333.1)**	GH43	Bacterium	*Lactobacillus brevis* ATCC 367	-	-	2.96±0.24	-	407±9	137.5	[36]
**BsX (CAB13642.2)**	GH43	Bacterium	*Bacillus subtilis subsp*. *subtilis* str. 168	-	-	2.25±0.05	-	128±1	56.89	[36]
**AmX (ABR49445.1)**	GH43	Bacterium	*Alkaliphilus metalliredigens QYMF*	-	-	1.44±0.14	-	56.9±2.0	39.51	[36]
**SXA (ABR49445.1)**	GH43	Bacterium	*Alkaliphilus metalliredigens QYMF*	-	-	2.12±0.11	-	211±4	99.52	[36]
**β-D-xylosidase**	-	Bacterium	*Bacillus pumilus*	-	-	2.9	-	18.1	6.24	[37]
**Pae1263**	-	Bacterium	*Paenibacillus terrae* HPL-003	50	6.0	6.417	0.07576	-	-	[38]
**GbtXyl43A (DQ345777)**	GH43	Bacterium	*Geobacillus thermoleovorans* strain IT-08	-	5.0	12.8±0.93	-	0.065±0.002	0.005	[39]
**XylBH43 (BAB07402)**	GH43	Bacterium	*Bacillus halodurans*	-	-	3.02±0.78	-	117±10	38.74	[40]
**BxTW1 (KP119719)**	GH3	Fungus	*Talaromycesamestolkiae*	70	3.0	0.48 ±0.05	0.055±0.0013	183	381.25	[41]
**β-xylosidase (KIF125)**	-	Fungus	*Trichoderma asperellum KIF125*	60	4.0	0.14	-	32.3	230.71	[42]
**β-xylosidase**	-	Fungus	*Emericella nidulans*	55	4.5–5	1.0	-	992.4	992.4	[43]
**β-xylosidase**	-	Fungus	*Trichoderma viride*	55	3.5	2.1	-	36.4	17.33	[44]
**AnBX (AF108944)**	GH3	Fungus	*Aspergillus niger* ASKU28	70	4–4.5	30.8±3.7	-	67.8±7.9	2.20	[45]

* Optimum temperature

**Optimum pH.

## 4. Discussion

The rumen is a complex anaerobic environment. There the ingested food is fermented by microorganisms to a mixture of volatile fatty acids, which is the major nutritional source for the ruminant animal [46]. The rumen microbiota has evolved to hydrolyze plant biomass, and therefore it is a perfect environment for finding different enzymes involved in this process. The Caatinga is a semiarid biome in the Northeastern part of Brazil. Despite the many similarities of the rumen from different animals, the environment where the animal lives may influence its microbiota as well. For instance, uncultured archaeal sequences belonging to methanogens of “uncultured marine bacteria” groups II and III were identified in the Caatinga goat rumen [12]. The free ranging Caatinga goats from which samples were taken had access to high-salt water ponds, which may be the reason for the presence of these groups in the Moxotó goat rumen. A total of 16.4% of the solid fraction sequences from the goat rumen were from unclassified *Bacteria*. This fact highlights the presence of many unknown microorganisms, which make the Caatinga Moxotó goat rumen an interesting place for enzyme discovery.

Sequencing of microbial genomes followed by bioinformatics analysis is one approach to identify new candidate enzymes. However, this method has been criticized as it is biased towards related sequences. Functional screens of metagenomic libraries, which are based on enzymatic activity rather than sequence, are a powerful method to identify new enzymes. Regardless of the method used to initially identify a new enzyme, its detailed biochemical characterization is extremely important as enzyme properties (e.g., substrate specificity, optimum temperature) cannot be inferred from sequence, and practical applications are dependent on these biochemical properties.

A Moxotó goat rumen metagenomic library had been previously constructed [13], and here we report a functional screen of this library using esculin as substrate. Esculin consists of a glucose molecule bound to esculetin, which upon release through hydrolysis reacts with iron citrate from the medium forming a dark precipitate. The dark precipitate observed indicates a potential glucosidase enzymatic activity. Using this assay, a functional screen of 10,839 metagenomic library clones was performed, and yielded three confirmed positive clones. Of these, Clone 11 showed the strongest activity on the esculin plate assay, and was chosen to be completely sequenced.

*In silico* analysis of Clone 11 showed the presence of three ORFs; ORF1 and ORF2 being complete, and ORF3 being partial. BlastP analysis showed that ORF2, named *BGL11*, is 78% identical to a beta-glucosidase from a *Prevotellaceae bacterium* and 73% identical to a GH3 family carbohydrate active enzyme from *Prevotella* sp (FD3004). Bacteria from the genus *Prevotella* play important roles in rumen carbohydrate and nitrogen metabolism [46, 47]. *Prevotella ruminicola* strain 23 has been studied since its isolation in 1958. This species is not cellulolytic, but can efficiently breakdown hemicellulose and pectin. As expected, *P*. *ruminicola* 23 genome sequence showed a large number of GHs for hemicellulose depolymerization and utilization [48].

Because *BGL11* comes from an unknown bacterium from the family Prevotellaceae, the *BGL11* sequence was compared to the *Prevotella ruminicola* 23 complete genome sequence. BGL11 protein sequence is 69% identical to a GH3 encoding gene (PRU_2312); which has on both sides genes involved in carbohydrate metabolism: a GH2 coding gene (PRU_2313); an alfa-glucosidase (PRU_2314); a putative glycosyl hydrolase (PRU_2308), and a putative polygalacturonase/ beta-xylosidase (PRU_2307). Similarly, *in silico* analysis of the Clone 11 insert showed a cluster of three carbohydrate metabolism-related ORFs and no terminator (Fig 1). This genomic organization suggests that *BGL11* is part of an operon.

A more detailed analysis further suggests that the Clone 11 operon, which includes BGL11, is involved in hemicellulose degradation. This hypothesis is supported by the fact that the upstream region of *BGL11* is an ORF 76.32% identical to an alpha/beta hydrolase family protein from *Prevotella* sp. BP1-148, and also 76.32% identical to a CBM6 carbohydrate-binding protein from *Prevotella* sp. BP1-148. The CBM6 domain is usually appended to GH11 glycoside hydrolases or GH43 xylanase domains. The downstream region of *BGL11* presented similarity to two sequences: an endo-1,4-beta-D-glucanase Y from *Prevotella* sp. BP1-148 (69.15% identity) and a glycoside hydrolase, family 10, from *Rhodopirellula europaea* SH398 (39.23% identity). Therefore, both the upstream and downstream regions of BGL11 may contain genes involved in hemicellulose degradation.

Furthermore, seven conserved domains were found in BGL11 (Table 1), however, information is available for only a few of these. Three Pfam conserved domains were predicted to be present in the BGL11 protein (i.e., Glyco_hydro_3_C, FN3-like, and Glyco_hydro_3). Glyco_hydro_3_C is involved in catalysis, and may play a role in beta-glucan binding [49]. The conserved FN3-like module can be present in cellulosomes, independently or as part of another protein. Cellulosomes are large protein complexes that act on cellulolytic substrates. In this system, multiple enzymatic domains are linked to structuring proteins bound to the bacterial cell wall. Cellulosomes contain carbohydrate-binding modules (CBMs), glycoside hydrolases (GHs), and some domains of unknown function such as X-domains, X-modules, and fibronectin type 3-like (Fn3-like) modules [50, 51]. It has been hypothesized that Fn3-like domains work as a linker peptide to connect different modules of an enzyme which can be extended or they can be simply act as spacers between domains [52]. Other studies support the idea that this module can work as a cellulose disruptor improving the hydrolytic capability of cellulases [53, 54].

BGL11 has four other conserved domains (i.e., BglX, PRK05337, PRK15098, PLN03080). BglX modules are found in GH3 family periplasmic beta-glucosidases. BglX conserved domain includes a signal peptide that addresses the enzyme to the periplasmatic space. This corroborates the Signal-BLAST and SignalP5.0 results that predict a signal peptide in the BGL11 N-terminus. Conserved domain PRK05337 is related to the Glyco_hydro_3 conserved domain.

In this work, the native sequence for BGL11 was cloned into the pET21a(+) expression vector. For protein expression, the autoinduction protocol was used, resulting in BGL11 being expressed and secreted to the medium. Production of secreted heterologous proteins in *E*. *coli* increases the amount of expressed protein, and facilitates the purification step, reducing cost and saving time [55].

BGL11 enzyme specificity was tested using synthetic aryl substrates, a common practice in biochemical studies. The data obtained using these synthetic substrates suggested that BGL11 was a bi-functional β-glucosidase/β-xylosidase enzyme, with similar activities towards the substrates pNPG and pNPX. However, upon further investigation of BGL11 substrate specificity using natural substrates, activity towards xylobiose, cellobiose, salicin, and lactose was revealed. BGL11 activity is highest towards xylobiose. Therefore, these data suggest that BGL11 is a β-xylosidase with multiple substrate specificity. This result highlights the fact that testing enzyme activity towards natural substrates is fundamental. The use of synthetic substrates is very practical, but often misleading. Dodd, D. and I. K. O. Cann. (2009) [5] argued that the catalytic promiscuity exhibited by many GHs shows the limitations of artificial substrates to determine function of unknown enzymes. For industrial application of new enzymes, the use of natural substrates for biochemical characterization is crucial because these are the substrates enzymes will act upon. Other biochemical properties of BGL11 were also determined. BGL11 optimal pH of 5.6 is similar to those of other β-xylosidases from bacteria (Table 3); and higher than that of many β-xylosidases of fungal origin. The optimum temperature of 50°C suggests that BGL11 can be used in mesophilic industrial processes. Regarding enzyme stability, at 40°C purified BGL11 remained active for more than 15 hours (i.e., 900 minutes) without any significant reduction in activity. At 50°C, after 7 hours (i.e., 420 minutes) of incubation, BGL11 remained 60% active. A review of biochemical properties of β-xylosidases [56] places BGL11 enzyme stability as higher than that of many other β-xylosidases for which this parameter was studied. It is important to note that enzyme stability can be further increased at higher temperatures by formulation with additives such as salts, polyols and sugars. In future work, BGL11 may also be immobilized and/or engineered for even higher stability.

Most of the β-xylosidases characterized kinetically belong to the GH43 family, and characterization was performed using the synthetic substrate pNPX. A literature search identified 73 β-xylosidases whose kinetic parameters have been reported. Only 19 of these enzymes have been characterized using xylobiose in enzyme kinetic experiments, and these were shown on Table 3. For BGL11 the *K*_m_ was 3.88 ± 0.31 mM, the V_*max*_ 0.0385 ± 0.00084 mmol. min^-1^. mg^-1^, *K*_cat_ 57.79 s^-1^ and *K*_cat_/*K*_m_ 14.89 s^-1^. mM^-1^. To the best of our knowledge, BGL11 is the first bacterial GH3 β-xylosidase to have its kinetic parameters determined using the natural substrate xylobiose. There are two other GH3 β-xylosidases, BxTW1 and AnBX, which have also been characterized using xylobiose, but these are fungal enzymes [41, 45]. The *K*_m_ for BxTW1 and AnBX was 0.48 ± 0.05 mM and 30.8 ± 3.7 mM, respectively. Therefore, BGL11 has higher affinity towards xylobiose than AnBX, but a lower affinity than BxTW1. The BGL11 *K*_m_ is in a similar range to those of other six GH43 β-xylosidases on Table 3 (i.e., 2–3 mM). BGL11 also has a *K*_m_ similar to that of another metagenome derived GH43 enzyme, CoXyl43 (i.e., *K*_m_ = 2.02 ± 0.06 mM), but a higher *K*_cat_ (57.79 s^-1^ in comparison to 17.82 s^-1^). Regarding the constant that gives the kinetic specificity or efficiency of the enzyme (*K*_cat_/*K*_m_), for BGL11 this value is 14.89 s^-1^. mM^-1^. There are no other bacterium β-xylosidases from family GH3 available for comparison.

β-xylosidases such as BGL11 have several possible applications in industry [56]; some in association with cellulases, and others in association with other hemicellulases. For example, they can be used to promote maceration in industrial processing of vegetable fibers. In the wine industry, in association with other enzymes, β-xylosidases reduce β-glucans, thus decreasing must viscosity and losses in wine clarification; and similarly, in the beer production industry, beer viscosity and turbidity are also reduced. In the juice industry, β-xylosidases contribute in fruit juice extraction and clarification, thus improving process performance and product appearance. In the baking industry, β-xylosidases and other enzymes can be applied to flour to improve dough handling and shelf-life, as well as bread volume and crumb structure. When applied to animal feed, β-xylosidases in combination with other enzymes increase feed efficiency, saving costs and improving productivity. In a biorefinery, β-xylosidases can be used to release xylose from plant biomass so that it can be fermented into valuable bioproducts such as xylitol and ethanol.

## 5. Conclusions

The functional metagenomic approach is an interesting strategy to identify new enzymes. This is the first time that the Caatinga goat rumen environment was explored for enzyme discovery. The focus of the functional metagenomic approach is enzymes of bacterial origin. Our screen allowed the identification of BGL11 which is encoded by a gene from an uncultivated (unknown) bacterium. BGL11 is a novel β-xylosidase from GH3 family that carries a signal peptide sequence, which allowed it to be secreted by *E*. *coli*. BGL11 showed similar activity towards the synthetic substrates pNPX and pNPG. However, when tested with natural substrates, BGL11 showed 179-fold greater activity toward xylobiose than cellobiose. To the best of our knowledge, this is the first GH3 β-xylosidase of bacterial origin to be biochemically characterized using the natural substrate xylobiose. Many scientific studies use synthetic substrates to determine enzyme specificity, but these may not reveal the most relevant specificity. BGL11 also showed activity towards cellobiose, salicin and lactose; thus, it is a multifunctional enzyme. The BGL11 ability to hydrolyze different substrates may be an interesting property for its utilization in a range of potential industry processes. Its stability at 40°C and 50°C suggest it can be used in mesophilic industrial processes for plant biomass digestion.

## Supporting information

S1 Fig (TIF)

S2 Fig (TIF)

S1 File (PDF)
