# Supplementary figures and images for: Functional screening of a Caatinga goat (Capra hircus) rumen metagenomic library reveals a novel GH3 β-xylosidase
Source: PLoS One. 2021 Jan 15;16(1):e0245118. doi: 10.1371/journal.pone.0245118 (PMC7810302; doi:10.1371/journal.pone.0245118)

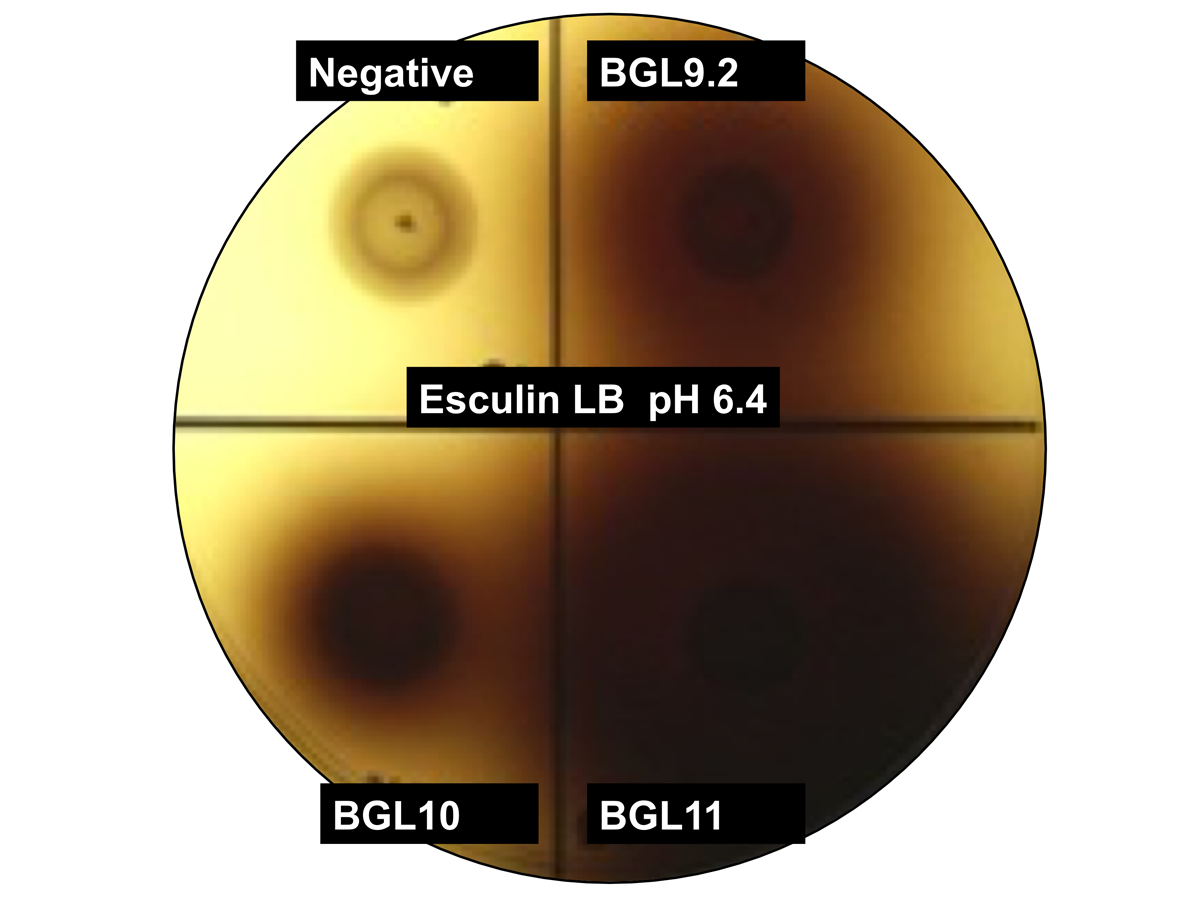

Supplement: S1 Fig — (TIF) [file pone.0245118.s001.tif]

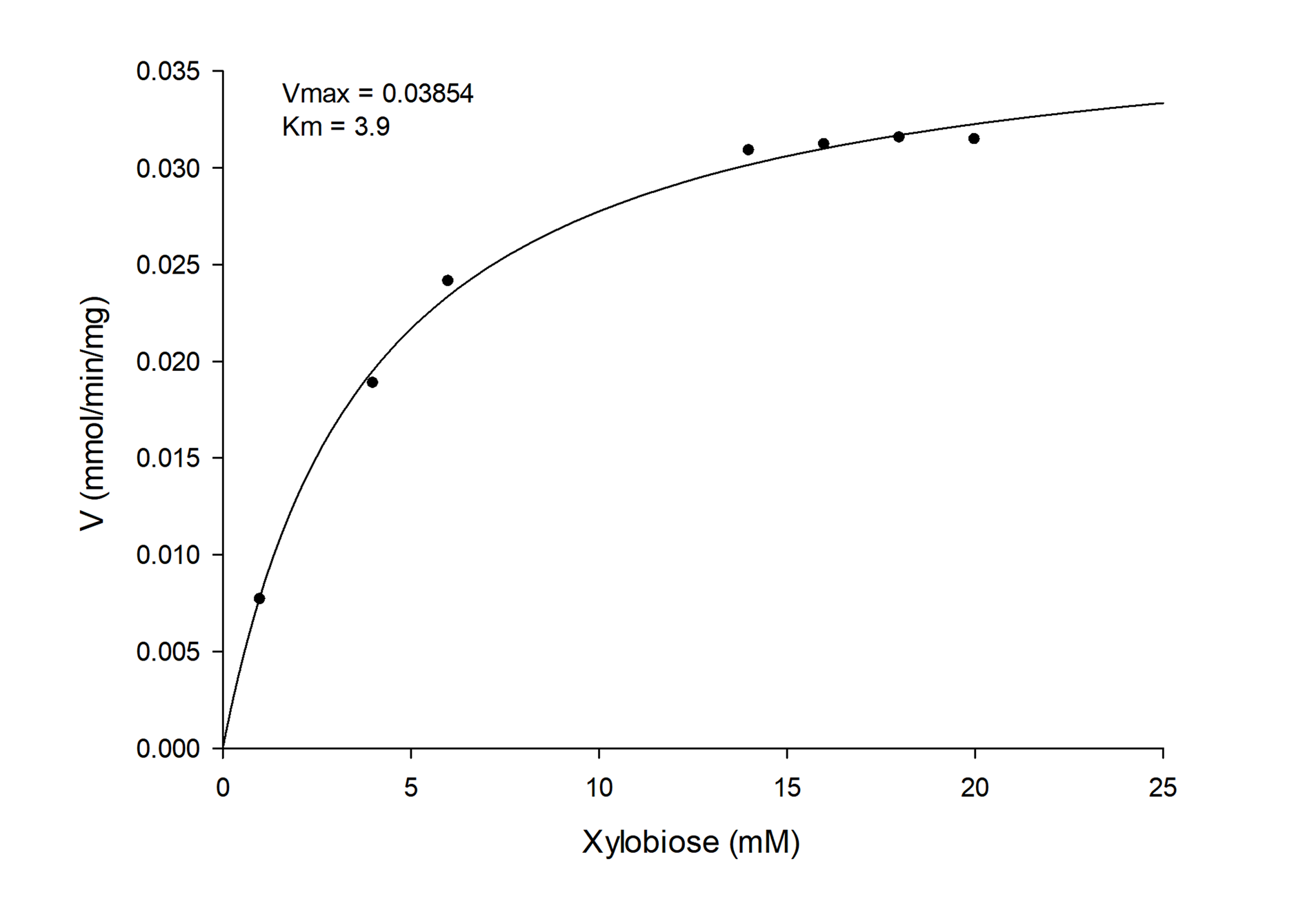

Supplement: S2 Fig — (TIF) [file pone.0245118.s002.tif]

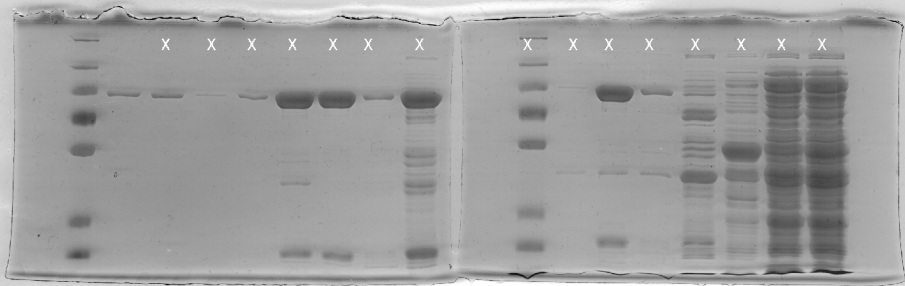

First two lanes were used in Fig.5. Photo taken with camera.

Supplement: S1 File — (PDF) [file pone.0245118.s003.pdf]
